# Manganese dioxide-coated biocarbon for integrated adsorption-photocatalytic degradation of formaldehyde in indoor conditions

**DOI:** 10.1016/j.heliyon.2024.e29993

**Published:** 2024-04-24

**Authors:** Mariem Zouari, Silvo Hribernik, Laetitia Marrot, Marian Tzolov, David B. DeVallance

**Affiliations:** a InnoRenew CoE, Livade 6a, 6310, Izola, Slovenia; b Faculty of Mathematics, Natural Sciences, and Information Technologies, University of Primorska, Muzejski trg 2, 6000, Koper, Slovenia; c Faculty of Electrical Engineering and Computer Science, University of Maribor, Koroška cesta 46, SI-2000, Maribor, Slovenia; d FRISSBE, Slovenian National Building and Civil Engineering Institute (ZAG), 1000, Ljubljana, Slovenia; e College of Science and Technology, Commonwealth University of Pennsylvania, 401 North Fairview Street, Lock Haven, PA, 17745, United States

**Keywords:** Biocarbon, Manganese dioxide, Catalytic degradation, Functional coating, Built environment

## Abstract

Formaldehyde is a common indoor air pollutant with hazardous effects on human health. This study investigated the efficiency of biocarbon (BC) functionalized with variable contents of MnO_2_ for formaldehyde removal in ambient conditions via integrated adsorption-photocatalytic degradation technology. The sample with the highest formaldehyde removal potential was used to prepare a functional coating made of acrylic binder mixed with 20 wt% of the particles and applied on beech (*Fagus sylvatica* L) substrate. SEM images showed that MnO_2_ was deposited around and inside the pores of the BC. EDX spectra indicated the presence of Mn peaks and increased content of oxygen in the doped BC compared to pure BC, which indicated the successful formation of MnO_2_. Raman spectra revealed that the disorder in the BC's structure increased with increasing MnO_2_ loadings. FTIR spectra of BC–MnO_2_ samples displayed additional peaks compared to the BC spectrum, which were attributed to MnO vibrations. Moreover, the deposition of increased MnO_2_ loadings decreased the porosity of the BC due to pores blockage. The BC sample containing 8 % Mn exhibited the highest formaldehyde removal efficiency in 8 h, which was 91 %. A synergetic effect between BC and MnO_2_ was observed. The formaldehyde removal efficiency and capacity of the coating reached 43 % and 6.1 mg/m^2^, respectively, suggesting that the developed coating can be potentially used to improve air quality in the built environment.

## Highlights

• Biocarbon from *Arundo Donax* was doped with MnO_2_ and used for formaldehyde remediation.
• The structural and morphological properties of biocarbon changed with the MnO_2_ loadings.
• Biocarbon-MnO_2_ effectively removed formaldehyde via adsorption and photodegradation.
• Biocarbon-MnO_2_ particles were used to prepare a functional coating for wood.
• The coating successfully removed formaldehyde in indoor conditions.

## Graphical abstract

**Figure:**
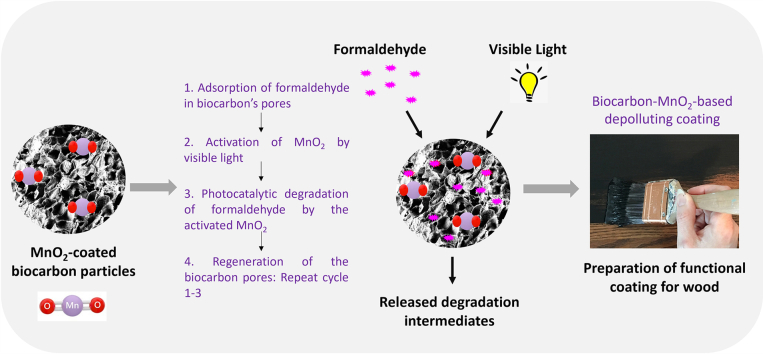

## 1 Introduction

The continuously growing environmental awareness and concerns about public health increase the focus on enhancing air quality. In this view, quests for clean indoor air have intensified. Volatile organic compounds (VOCs) are among the significant airborne pollutants detected indoors. VOCs occurrence indoors has been associated with causing sick building syndrome [1], which affects occupants’ well-being. VOCs thresholds in built environments are subject to strict regulations in many countries, specifically in the case of highly toxic compounds such as formaldehyde. Formaldehyde is a common indoor air pollutant and is classified as a group 1 carcinogen [2]. Exposure to formaldehyde was associated with serious diseases such as pulmonary problems [3], cancer [4], and asthma [5]. Given the adverse health effects of formaldehyde, different regulations and occupational health authorities have set a limit threshold of exposure. The acceptable exposure levels were concluded based on outcomes from toxicological and epidemiological assessments. For instance, the World Health Organization (WHO) [6] suggested not exceeding 100 μg/m^3^ of formaldehyde during a 30-min short-term exposure period. For long-term exposure, no safe limits were specified. Some legislation further decreased the maximum allowed safe level of exposure. For example, the Official Journal of the French Republic (JORF) indoor guidelines [7] adopted a maximum long-term exposure limit of 10 μg/m^3^ from 2023, noting that the previous limit that was considered in the period between 2016 and 2022 was 30 μg/m^3^. Formaldehyde levels were investigated in several European countries and in different built environments. Cases where relatively high formaldehyde levels were detected are listed in Table 1.

**Table 1:** Examples of cases where high formaldehyde levels were detected indoors.

Country	Building type	Formaldehyde concentration, μg/m^3^	Reference
France	Dwellings	>10	[8]
France	School classrooms	15.9	[9]
Italy	Printing facilities	25.8	[10]
Spain	Living rooms	17.1 to 91.4	[11]

The excess of formaldehyde levels, in some cases with the increased public awareness about the health risks of formaldehyde, is driving demand for enhanced air purification methods. Several methods have been proposed to mitigate indoor formaldehyde levels, including adsorption. Adsorption has gained a good reputation, being one of the simplest yet efficient techniques for removing gaseous pollutants. However, as a non-destructive technique, adsorption is limited by saturation, which occurs when the adsorbent's pores are filled with the pollutant's molecules. Alternatively, photocatalytic oxidation technology efficiently removes gaseous molecules at relatively low concentrations [12]. This method is based on applying photocatalysts, which are semiconductor materials targeted toward degrading various contaminants, including VOCs [13]. In the presence of sufficient energy, the photocatalysts convert pollutants by oxidation or reduction into less harmful intermediates (H_2_O and CO_2_) [14]. However, the low surface area of photocatalysts is usually a burden against sufficient capturing of VOCs from the air phase to their surface to start the degradation process. The combination of adsorption and photocatalytic degradation has been viewed as a promising technique for air purification. In this process, the VOCs in the bulk air phase are captured on the porous adsorbent and then degraded by the photocatalytic particles deposited on the adsorbent. In this case, the adsorption sites are freed, and more VOCs can be trapped in the adsorptive material. Selecting an adequate adsorbent and photocatalyst combination is essential to achieve maximal efficiency.

Owing to the high porosity and large surface area, biocarbon (BC) has been efficiently used for the adsorption of pollutants, including VOCs [15,16]. BC is an environmentally friendly material that can be easily prepared from a wide range of feedstocks via a slow pyrolysis process. A well-developed porous structure and high affinity for VOCs make BC a suitable platform for immobilizing active photocatalysts. The BC will capture VOCs onto its structure. Then, the immobilized photocatalyst will act and degrade the captured pollutants [17]. The synergy between BC and the photocatalyst can create an efficient adsorption-photodegradation system.

Manganese dioxide (MnO_2_) has been viewed as an efficient visible light photocatalyst with a relatively narrow band gap energy (1–2 eV) [18]. MnO_2_ was widely used for formaldehyde removal, specifically at relatively elevated temperatures. For instance, total formaldehyde degradation was achieved by MnO_2_ at 75 °C [19], 80 °C [20], and 100–225 °C [21]. Nevertheless, the efficiency of MnO_2_ is limited at ambient temperatures as it reaches the deactivation state faster, making it unsuitable for application in indoor environments. Li et al. [22] reported that formaldehyde removal decreased from 75 % to 10 % after 3 h at ambient temperature when evaluating MnO_x_ supported on granular activated carbon via the in situ reduction method. The structure of MnO_2_, specifically the presence of tunnels (i.e., channels) in the internal structure, determines its catalytic efficiency [23,24]. A 2D tunnel structure is favourable for better diffusion and conversion of pollutants compared to 1D structure [23]. Therefore, selecting the appropriate preparation route is key to improving the catalytic ability of MnO_2_ in indoor conditions. Zhang et al. [25] synthesized a lignocellulose-based activated carbon fiber paper loaded with MnO_2_ and observed a decrease in formaldehyde concentration of 59 ± 6 ppm after 10 h at room temperature. Similarly, Dai et al. [23] prepared δ-MnO_2_ deposited on activated carbon fibers using the co-precipitation method. They found that the sample's ability to degrade formaldehyde was 97 % after 10 h at 25 °C. Other studies [23,25] successfully used MnO_2_ to remove formaldehyde at ambient temperature. However, the initial formaldehyde concentrations used for testing were relatively high (150 ppm or above), which would not be common within built environments. Thus, the investigation of the photocatalytic degradation potential of MnO_2_ at ambient conditions and in the presence of low formaldehyde concentrations seems to be interesting, considering that relevant research is still limited, specifically, the combination of MnO_2_ with BC. Further exploitation of the BC–MnO_2_ hybrid particles for developing novel materials, such as functional coatings, with air purification ability can pave the way towards realistic application in the indoor environment. Indeed, depolluting coatings have emerged as promising materials that possess conventional surface protection role beside the ability to improve air quality. Coatings usually cover a large surface area in the built environment, which increases the interaction with pollutants in the air and enables high removal efficiency. The novelty of the current study consists of developing a coating with dual function for indoor formaldehyde removal which can open perspectives for using BC–MnO_2_ particles in coatings applications.

The objectives of this study were to (i) prepare BC particles loaded with variable concentrations of MnO_2_ and investigate their formaldehyde removal efficiency in indoor conditions and (ii) utilize the BC–MnO_2_ particles for the preparation of functional coating for formaldehyde remediation.

## 2 Materials and methods

### 2.1 Materials and reagents

KMnO_4_ and MnSO_4_ (analytical pure reagents) were purchased from Sinopharm Chemical Reagent Co., Ltd and used as received. Biocarbon (BC) was prepared from demineralized *Arundo donax* biomass via slow pyrolysis at 800 °C for 30 min. Details about the preparation process and characterization of the BC are presented in [16]. Aqueous formaldehyde solution (37 % w/v) was purchased from Carlo Erba reagents (Dasti group, Val de Reuil, France). Waterborne acrylic (UNIKOM for wooden surfaces) was provided by Samson company (Kamnik, Slovenia) and used as a binder for the coating preparation. High-purity nitrogen and CO_2_ gases (Grade 5.0) were used for physisorption analysis. Beech (*Fagus sylvatica* L) substrates (13 cm × 10 cm × 1 cm) were used as surfaces for the application of the coatings.

### 2.2 Synthesis of MnO_2_ and BC–MnO_2_ particles

MnO_2_ particles were prepared following the co-precipitation method reported by [23] and described as follows: 3.16 g KMnO_4_ and 18.12 g MnSO_4_ were mixed separately with 50 mL of distilled water each and dissolved by stirring at 80 °C for 20 min. Subsequently, the KMnO_4_ and MnSO_4_ solutions were added dropwise into 50 mL of distilled water, respectively. The obtained mixture was then kept under continuous stirring at 80 °C for 2 h to enable the reaction and formation of MnO_2_. The precipitate was then collected after filtration and washing and left to dry in the oven at 80 °C overnight.

To prepare BC-supported MnO_2_, 2 g of BC powder was mixed with 50 mL of an aqueous solution of MnSO_4_. KMnO_4_ solution (50 mL) was then added dropwise. The mixture was vigorously stirred at 80 °C for 2 h. The obtained product was filtered, washed, and oven-dried at 80 °C. The amounts of KMnO_4_ and MnSO_4_ were varied to achieve variable MnO_2_ loadings on the BC material. Four different BC–MnO_2_ batches were prepared. The samples were named “BC–MnO_2_-x,” where BC refers to biocarbon and x refers to the sample's number. Details about the different formulations are provided in Table S1 in the supplementary materials.

### 2.3 Characterization of the BC, MnO_2_, and BC–MnO_2_ particles

The surface morphology of the samples was evaluated using a field emission scanning electron microscope Carl Zeiss SUPRA 35 VP. Powder samples were attached to SEM sample holders via double-sided carbon adhesive tape and sputtered with a thin layer of gold (Denton Vacuum LLC). Imaging was performed at an accelerating voltage of 1 kV and a working distance of approx. 4.5 mm. Energy dispersive X-ray (EDX) analysis was employed to assess the elemental composition of the samples using a detector SDD Ultim max 100 (Oxford Instruments) and Aztec software. Elemental mappings were performed at 20 kV and at a 8.5 mm working distance.

The crystallographic structure was evaluated using a Bruker D2 Phaser diffractometer (Cu–Kλ radiation; 1.5406 Å). Measurements were performed with a step size of 0.03° and a time/step of 1 s. Samples were dispersed in isopropanol, deposited on a Si zero background sample holder, and dried to obtain a thin layer of particles. XRD spectra were collected in the 2θ range from 5° to 80°.

Raman spectroscopy was performed using a Bruker Senterra Confocal Raman Microscope. Spectra were recorded using a 532 nm laser beam, 0.2 mW power, 2 mm aperture, 20× Raman microscope objective, exposure time of 2× 10 s, and 3–5 cm^−1^ resolution. The laser power, exposure time, and objective were selected to avoid any visible modification of the material during these measurements. Spectra were processed using OPUS software (Bruker, Germany).

The surface functional groups were investigated using a Fourier transform infrared (FTIR) spectrometer (Alpha FT-IR Spectrometer Bruker, Billerica, MA, United States) connected to an ATR (attenuated total reflection) module. FTIR spectra were collected in wavelength ranges from 400 to 4000 cm^−1^, and a resolution of 4 cm^−1^. For each sample, 64 scans were performed. Spectra were further treated by eliminating CO_2_ and atmospheric water vapor effects. All samples were tested before exposure to formaldehyde. Moreover, the BC–MnO_2_ sample with the highest formaldehyde removal efficiency was also tested after exposure to formaldehyde to detect eventual changes in surface functional groups.

The porosity of the samples was evaluated by physisorption analysis (Autosorb iQ-XR-AG-AG, Anton Paar Quantachrome Instruments, Florida, USA). Nitrogen gas was used to determine the specific surface area and total pores volume according to the BET model, while the pores distribution was determined according to the BJH model. CO_2_ gas was used to determine the microporous surface area and micropore volume according to the DFT model.

### 2.4 Adsorption-photocatalytic degradation test

The integrated adsorption-photocatalytic degradation activity of the MnO_2_, BC, and BC–MnO_2_ samples was tested in a closed glass chamber (25 L of volume) [16]. For each test, 1 g of sample was placed in a Petri dish and placed in the chamber. The test chamber was equipped with an electrochemical formaldehyde gas sensor (Stox-HCHO, EC Sense, Schäftlarn, Germany) with 0.1 ppm resolution and 1 s response time to detect the changes in formaldehyde concentration. A portable infrared sensor (JD-3002, JLDG, Guangdong, China) was used to investigate the eventual production of CO_2_ as a co-product of formaldehyde degradation. Then, 8 μl of formaldehyde solution was injected, and the chamber was hermetically sealed. The initial formaldehyde concentration was 4 ppm. The concentrations of formaldehyde and CO_2_ were continuously recorded for 8 h, noting that the experimental time was selected based on daylight availability. Experiments were performed in ambient conditions: temperature of 23 °C, relative humidity 40 %, and under visible light irradiation of 12 W LED lamp. One BC–MnO_2_ sample with the highest formaldehyde removal efficiency was also tested in dark conditions for comparison. The samples' formaldehyde removal efficiency (%) was determined based on the initial and residual concentrations in the test chamber using Equation (1).

(1) $$\text{Formaldehyde}\;\text{removal}\;\text{efficiency}=\frac{C_{i}-C_{f}}{C_{i}}\times 100$$

Where *C*_*i*_ is the initial formaldehyde concentration (ppm), and *C*_*f*_ is the final formaldehyde concentration after 8 h of the experiment (ppm).

A blank test was performed by repeating the same experiment without the presence of a sample. The reduction in formaldehyde concentration obtained for the blank was subtracted from the values obtained for each sample for results correction. Measurements were repeated three times for each sample, and average results were reported. The results were compared by one-way ANOVA analysis using Statistica 12.0 software.

The BC–MnO_2_ sample with the highest formaldehyde removal efficiency was selected for the reusability test. The formaldehyde removal efficiency was determined after five successive cycles without regeneration in between.

### 2.5 Preparation and evaluation of the coating

The BC–MnO_2_ sample with the highest formaldehyde removal efficiency was utilized to evaluate its potential as an additive in a coating application. The waterborne acrylic binder was loaded with 20 wt% of BC–MnO_2_ powder and mixed manually. During preparation, a maximum of 20 wt% of BC particles could be added to the acrylic base while keeping a texture that allows brushing on a surface. The mixture was then applied on a beech substrate by brushing, followed by drying for 2 h at room temperature. We did not observe any weight loss of the coating due to BC–MnO_2_ particles but rather by water loss due to evaporation as the acrylic component cured. The final thickness of the coating was measured by microscopic cross-sectional observation using a digital microscope Keyence VHX-6000 (Keyence corporation, Itasca, IL, USA). A reference sample, which consisted of wood coated with pure binder (0 wt% BC–MnO_2_), was also prepared. The formaldehyde removal efficiency of the coatings was evaluated using the same method and equation described above in section 2.4. Aluminum tape was used to cover the uncoated parts of the samples (edges and bottom surface) to prevent their interaction with the formaldehyde molecules. Samples were stored in a desiccator until the formaldehyde removal test was performed to prevent moisture absorption from the atmosphere or deposition of dust particles, which can affect the accuracy of the results. The formaldehyde removal capacity (Q) (mg/m^2^) per coated surface area was determined using Equation (2).

(2) $$Q=\frac{\left(Ci-Cf\right)\times V}{SA}$$

Where *C*_*i*_ is the initial formaldehyde concentration (mg/L), *C*_*f*_ is the final formaldehyde concentration after 8 h of the experiment (mg/L), V is the volume of the test chamber (L), and SA is the surface area of the coating (m^2^).

## 3 Results and discussion

### 3.1 Surface morphology and elemental composition of the samples

**Fig. 1:**
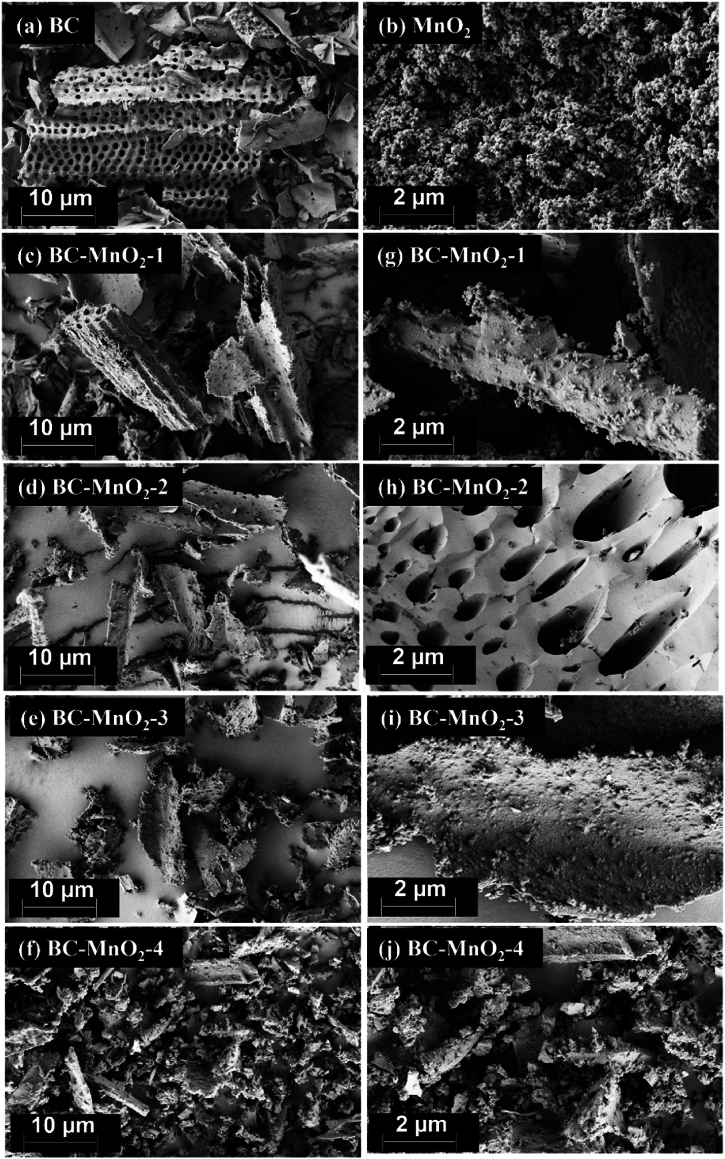
SEM images of (a) BC, (b) MnO_2_, (c–f) BC–MnO_2_ at 10 μm, and (g–j) BC–MnO_2_ at 2 μm.

The morphology of the biocarbon (BC), MnO_2_, and BC–MnO_2_ samples was observed by SEM at different magnifications, and the images obtained are represented in Fig. 1.

The porous structure of the BC sample was observed in the SEM image (Fig. 1a). The pores generally exhibited an organized round shape. The porous structure of BC makes it an ideal platform for the deposition of MnO_2_ particles. The MnO_2_ (Fig. 1b) appeared as aggregates of spherical nanoparticles. The BC–MnO_2_ samples were observed as irregular BC pieces with rough surfaces, as compared to non-coated BC sample, indicating adsorbed particles deposited during the in-situ co-precipitation of MnO_2_ in the presence of BC particles (Fig. 1c–f). Images of the BC–MnO_2_ at a higher magnification (Fig. 1g–j) enable even closer inspection of the samples’ surface topography, most prominently revealing layers of densely packed MnO_2_ particles with scattered larger aggregates of oxide particles on the surface and inside the pores (Fig. 1h) of the BC, indicating that the doping process was successful. Small aggregates of MnO_2_ were distributed onto the BC structure. Choi et al. [26] reported similar observations of MnO_2_ particles deposited on activated carbon. Likewise, [27] found that the MnO_2_ particles were condensed on the BC surface and formed 30 nm in diameter aggregates. It is important to note that regardless of the amount of manganese precursors used (i.e., obtained degree of particle deposition), none of the BC–MnO_2_ samples have had their morphology drastically altered, which might be the case if thicker layers of particles are applied; thus indicating that indeed nano-sized layers are enveloping the BC particles in our case.

**Fig. 2:**
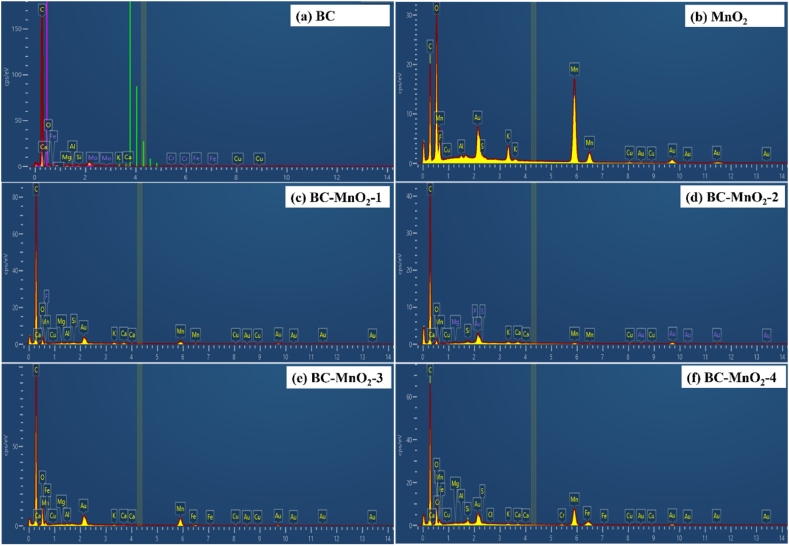
EDX spectra of (a) BC, (b) MnO_2_, (c–f) BC–MnO_2_.

EDX spectroscopy was used to examine the elemental composition of the samples and visualize the spatial placement of given elements, all of which also corroborate findings from the SEM micrographs. The EDX spectra are represented in Fig. 2. The weight percentages of carbon (C), oxygen (O), and manganese (Mn) are summarized in Table 2. The other elements category includes all remaining components that were detected in minor concentrations, such as Mg, Si, K, Cu, Ca, etc. These elements can originate from the ash fraction in the BC and the precursors (KMnO_4_ and MnSO_4_) utilized to prepare the MnO_2_.

**Table 2:** Elemental composition of the different samples.

Sample ID	Elemental composition, wt(%)				
	C	O	Mn	Other elements	Total
BC	91	6	0	3	100
MnO_2_	31	28	31	10	100
BC–MnO_2_-1	82	10	4	4	100
BC–MnO_2_-2	84	11	2	3	100
BC–MnO_2_-3	72	19	8	1	100
BC–MnO_2_-4	64	18	13	5	100

The BC sample had a relatively high C content, which was expected given that the utilized BC was prepared at 800 °C. However, O content was only 6 %. Indeed, a high pyrolytic temperature favors the degradation of biomass and the release of O and H in the form of volatiles. The obtained values (Fig. 2) were lower compared to values reported previously [27] for BC prepared from different types of biomass (miscanthus, switchgrass, corn stover and sugarcane bagasse) at 800 °C. It was found that weight percentages for C and O elements ranged between 73 % and 84 % and between 5 % and 11 %, respectively. The difference in findings was attributed to the differences in original biomass composition and the different methods used to assess the samples' composition. The EDX spectrum of the BC sample did not display any peak for the Mn element. However, a well-distinguished Mn peak was detected in the spectra of hybrid particles (BC–MnO_2_). The spectra showed an increase in the Mn peak with the increase in MnO_2_ loading, and BC–MnO_2_-4 had the highest Mn content (13 %). However, the Mn content of the BC–MnO_2_-2 sample was out of the trend and was lower than BC–MnO_2_-1. This anomalous result can be attributed to the heterogeneous distribution of MnO_2_ particles within the BC. As discussed in the SEM section, we observed two distinct morphologies of MnO_2_ populating the surface (and interior) of BC particles, including a continuous thin layer of nano-sized oxide particles and their discrete larger aggregates randomly attached to the surface. The distribution of Mn elements can be seen in the elemental mapping images (Fig. S3). Depending on the area of EDX inspection, either feature can be prevalent, with larger aggregates contributing to a greater extent to the Mn content, as opposed to a thin layer of nano-sized MnO_2_ particles. Indeed, EDX measurements were performed on randomly chosen areas of the samples, and their non-homogeneous surface topography can lead to some errors. Another important aspect in EDX analysis is interaction volume, defined as the region of the sample affected by the incident electrons, which results in the excitation of X-rays. To this end, MnO_2_ particles located beyond the confines of the interaction volume, that is, deeper within the samples' inner pores, will not contribute to the overall detected manganese content. Given the porosity of BC samples (Fig. 1) we can assume that a portion of manganese precursor solutions, which penetrated deeper into the BC particles' interior, resulted in the co-precipitation of oxide particles, which are trapped inside the BC and may be inaccessible to a certain extent for detection. Very large BC particles (cca. 10 μm) present in our samples can pose a challenge since the typical interaction volume at the accelerating voltage used would be a few microns, which would also present the limit of the achieved spatial resolution. One final point in regards to the EDX microanalysis, which touches more on the technical aspect of the execution of analysis itself, is the roughness of the sample: while macroscopically smooth, prepared particle layers exhibit high microscopical roughness, with particles scattered and tilted at different angles – these features can result in shadow areas which can in turn also interfere with the signal as it travels to the detector. The C content decreased from 91 % to 64 % for pure BC and BC–MnO_2_-4, respectively. However, the O concentration followed a similar trend to the Mn, suggesting that MnO_2_ particles were formed. El-Shafie et al. [28] prepared pistachio shells-derived BC doped with TiO_2_ and evaluated the particles' efficiency in adsorptive removal and photocatalytic decolorization of methyl orange. They obtained similar results from the EDX analysis. They reported that for BC doped with 1 %, 2 %, and 3 % of TiO_2_, the C concentrations decreased gradually while the O and Ti concentrations increased. Results were attributed to the successful formation of TiO_2_ particles on the BC.

Other elements were also detected in all samples (Fig. 2), which could originate from the inorganic fraction of the BC and Au, coming from the sputtered layers and Al, originating from the specimen holder stubs; these elements were removed from the analysis. Another study [29] stated that Mg and K peaks detected in coffee ground-derived BC originated from raw biomass.

### 3.2 Structural properties and surface functional groups of the samples

**Fig. 3:**
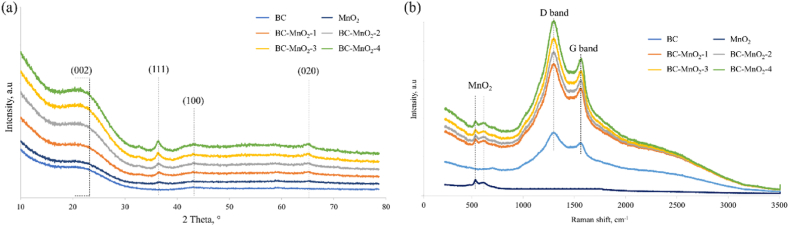
(a) XRD patterns and (b) Raman spectra of BC, MnO_2_, and BC–MnO_2_ samples.

The XRD patterns of BC, MnO_2_, and BC–MnO_2_ samples are represented in Fig. 3a.

The BC diffractogram showed a broad peak at 22.5°, which was attributed to (002) plane and a slightly visible peak at 43.8° corresponding to (100) plane of carbon structures. The observed peaks are characteristics of graphite elements [25,30]. The MnO_2_ diffractogram displayed two peaks centered at 37.4° and 66.5°. These peaks were attributed to (111) and (020) planes, respectively, based on the JCPDS 80–1098 card. Prior studies [23,25,31,32] attributed similar peaks to type δ-MnO_2,_ also known as birnessite type MnO_2_. The diffractograms of the hybrid particles (BC–MnO_2_) displayed all the peaks observed in the MnO_2_ and the BC samples, suggesting that MnO_2_ was successfully loaded on the BC particles, as confirmed by EDX analysis. It is worth noting that the BC peaks (22.5° and 43.8°) were also present in the MnO_2_ diffractogram, suggesting that these peaks could be assigned to the testing substrate and not to the detection of carbon. Indeed, the disordered phase has low diffraction intensity, so the disordered carbon material may not be seen in the XRD diffractograms. However, Raman spectroscopy can be more sensitive to investigating the degree of structural order of carbon elements in the BC-containing samples and developing graphitic structures in the presence of MnO_2_. Fig. 3b represents the Raman spectra collected from the different samples.

The Raman spectra of the BC displayed two peaks at 1308 cm^−1^ and 1573 cm^−1,^ corresponding to the D band and G band, respectively. In carbonaceous materials, the D band is associated with disordered structures such as defects, distortions, and edges [33]. In other words, the D band results from defects in the lattice that cause broken symmetry of the hexagonal structure of the carbon atoms [34]. Meanwhile, the G band is associated with sp^2^-bonded carbon corresponding to well-organized graphitic structures [35]. The visible D and G bands signified that the BC material contained ordered and disordered structures. However, the D band was greater than the G band, suggesting that the sample structure tended to be more like a disordered type. The distinguished D band can likely be related to aromatic carbon structures usually arising at high pyrolysis temperatures, given that the BC utilized in this study was prepared at 800 °C. In this regard, a prior study [36] reported that the D band was visible in hemp-derived BC prepared at 600 °C or above, which correlated with the increased aromaticity of the samples.

The Raman spectra of MnO_2_ exhibited two peaks at 551 cm^−1^ and 621 cm^−1,^ which correspond to the manganese oxide lattice. Peaks at 634 cm^−1^, 640 cm^−1^, 569 cm^−1^, 576 cm^−1^, and 638 cm^−1^ were reported for MnO_2_ materials [[37], [38], [39]]. The variation in the positions of the peaks obtained for MnO_2_ depends on the crystal structure (E.g., α-MnO_2_, δ-MnO_2_, β-MnO_2_) [39]. Raman spectra of the hybrid samples (BC–MnO_2_) displayed all the peaks corresponding to the MnO_2_ and the BC materials.

The intensity ratio of the D band relative to the G band ID/IG (Table S2) was determined to investigate the defect level after the incorporation of MnO_2_ into the BC. The ID/IG ratio of all BC–MnO_2_ samples was higher than that of the BC sample. The change in the intensity ratio (Table S2) indicated structural changes in the BC upon adding MnO_2_. The increase in the ID/IG ratio suggested an increase in the defect degree, which can be attributed to the formation of MnO_2_ particle clusters within the BC structure. Similarly, a prior study [40] reported an ID/IG ratio increase when β-FeOOH/Fe_3_O_4_ nanoparticles were added to BC. They assigned this increase to the reduction in the carbon sp^2^ domain and justified that the β-FeOOH/Fe_3_O_4_ nanoparticles formed and grew on the graphitic structure of the material. The analysis of the Raman spectra revealed that the BC–MnO_2_ tended to have a defective structure, likely favored by the successful deposition of MnO_2_ onto the BC surface and possibly penetrating into the BC pores.

**Fig. 4:**
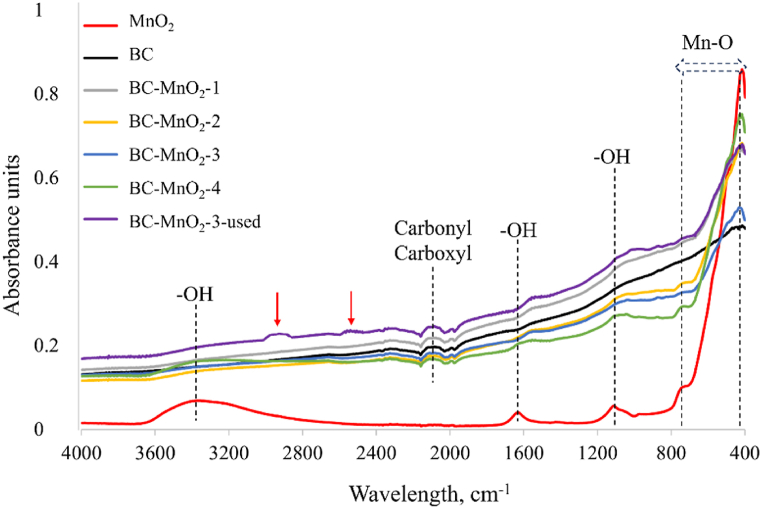
FTIR spectra of BC, MnO_2_, BC–MnO_2_.

The surface functional groups in the BC, MnO_2_, and BC–MnO_2_ samples were evaluated by FTIR analysis, and spectra are depicted in Fig. 4.

The broad peak between 3200 and 3600 cm^−1^ and the peaks at 1620 cm^−1^ and 1060 cm^−1^ correspond to O–H stretching and bending vibrations of hydroxyl groups, respectively, which was attributed to moisture [27,[41], [42], [43]]. The band between 2050 cm^−1^ and 2200 cm^−1^ was observed in BC and BC–MnO_2_ samples, while it was absent in MnO_2_. This band was attributed to the occurrence of carbonyl and carboxyl groups (Agin, 2013) associated with variable carbon structures (aromatic, carboxylic, and carbonyl carbon). These structures are more visible in spectra of BC prepared at high temperatures, which was the case in the present study (800 °C). Indeed, previous research [16] revealed that the band between 2050 cm^−1^ and 2200 cm^−1^ became visible in FTIR spectra of BC prepared at above 700 °C while it was not detected in spectra of BC prepared at 300 °C–600 °C. The bands below 750 cm^−1^ (between 700 and 400 cm^−1^) were detected only in MnO_2_ and BC–MnO_2_ samples and were assigned to Mn–O vibrations [44]. Results from FTIR suggested that MnO_2_ was successfully deposited on the BC particles.

### 3.3 Evaluation of the samples’ porosity

A combined evaluation of the samples was provided using nitrogen and CO_2_ at 77 K and 237 K, respectively, which provided a better understanding of the porosity (macro and mesopores) and microporosity (micropores) of the samples according to the recommendation of the International Union of Pure and Applied Chemistry (IUPAC).

Nitrogen adsorption-desorption isotherms and the pore size distributions of the different samples are represented in Fig. S2. Nitrogen isotherm of the BC displayed a type I isotherm according to the IUPAC classification [45], which is characterized by a relatively high nitrogen uptake at low pressure, indicating the microporous structure of the sample. The BC isotherm also had a non-reversible desorption branch (i.e., separated adsorption-desorption branches that do not close at low relative pressure). This feature can be commonly observed in the case of carbonaceous materials with microporous structures [46,47]. However, the MnO_2_ isotherm displayed a type IV shape with an H3-type hysteresis loop, indicating the dominance of mesopores in the structure[45,48].

The hybrid samples (BC–MnO_2_) displayed similar isotherm shapes, which consisted of hybrid type I/IV but with different nitrogen uptake. Type I was inherited from the BC's microporous feature, resulting in nitrogen uptake at low pressure. Type IV, accompanied by the presence of the hysteresis loop, was likely due to the incorporation of MnO_2_ and signified the occurrence of mesopores. The BJH pores size distribution (Fig. S2b) showed that the mesopores for all samples were concentrated in the region between 3 nm and 5 nm in width and that BC–MnO_2_ samples had higher mesopores content as compared to the BC regardless of the MnO_2_ loading.

The adsorption of nitrogen at cryogenic temperature (77 K) is usually limited due to kinetic restrictions (i.e., limited mobility of nitrogen molecules at 77 K). Thus, the CO_2_ adsorption-desorption isotherms were obtained at 273 K to evaluate the microporosity, given that CO_2_ molecules can access the narrow micropores. CO_2_ adsorption-desorption isotherms and the micropore size distribution of the different samples are represented in Fig. S3.

The CO_2_ adsorption-desorption isotherms showed that the BC and BC–MnO_2_ samples had a well-developed microporosity reflected by a relatively high CO_2_ uptake that ranged between 55 and 60 mmol/g. The MnO_2_ was the least microporous among all samples, with a CO_2_ uptake of only 11 mmol/g. The micropore size distribution (Fig. S3b) showed that the narrow pores were abundant in the region between 0.3 nm and 1 nm in width.

The specific surface area (BET), total pores volume, microporous surface area, and micropores volume of the samples are represented in Table 3.

**Table 3:** Physisorption analysis results.

Sample ID	Nitrogen		CO_2_	
	BET surface area, m^2^/g	Total pores volume, cm^3^/g	Microporous surface area, m^2^/g	Micropores volume, cm^3^/g
BC	291.400	0.190	587.175	0.151
MnO_2_	73.063	0.567	117.114	0.030
BC–MnO_2_-1	320.065	0.276	614.704	0.160
BC–MnO_2_-2	308.756	0.363	621.655	0.165
BC–MnO_2_-3	267.071	0.278	589.014	0.151
BC–MnO_2_-4	221.785	0.241	582.342	0.136

The BC sample had a relatively high BET and microporous surface area (Table 3), which was foreseen from the adsorption-desorption isotherms (Fig. S2 and Fig. S3). The BC sample was prepared at 800 °C, which resulted in a microporous character due to the high pyrolytic temperatures. The MnO_2_ had a relatively low BET surface area. The obtained value (73.063 m^2^/g) was comparable to a prior study [23] that reported 70.11, 74.84, and 84.50 m^2^/g for α-MnO_2_, γ-MnO_2_, δ-MnO_2_, respectively. MnO_2_ exhibited the highest total pores volume and the lowest micropores volume (Table 3), which confirms that MnO_2_ had a mesoporous structure as observed in the nitrogen and CO_2_ isotherms (Fig. S2 and Fig. S3).

The BET surface area of the BC increased slightly with the incorporation of MnO_2_ for BC–MnO_2_-1 and BC–MnO_2_-2. The increase in porosity was attributed to the increase in the number of mesopores brought by the MnO_2_, which was reflected by the improved total pores volume (Table 3). Cuong et al. [49] also reported that the BET surface area and mesopores volume of rice husk-derived BC increased after doping with MnO_2_ particles. Similarly, Zhou et al. [50] found that the BET surface area of BC increased by 32 % after deposition of MnO_2_ nanoparticles (19 %). They explained that the MnO_2_ formed spherical shape aggregates on the BC's surface, which enhanced the material's surface area. However, further increase in the MnO_2_ loadings (BC–MnO_2_-3 and BC–MnO_2_-4) led to a decrease in the BET surface area, which was likely related to the excess deposition of MnO_2_ particles inside and around the BC's pores as observed in SEM images (Fig. 1) leading to partial blockage. Similar observations were reported by Purwaningsih et al. [51] for MnO_2_-loaded graphene oxide. They explained that MnO_2_ nanoparticles were eventually inserted into the gaps between the carbon sheets. The microporous surface area followed the same trend as the BET surface area (Table 3).

The effect of MnO_2_ loadings on the BC's porosity should be considered when optimizing the doping process. Specifically, a balance needs to be obtained between the sample's porosity and the MnO_2_ content to ensure optimal adsorption and photocatalytic degradation performance.

### 3.4 Formaldehyde removal efficiency

#### 3.4.1 Formaldehyde removal by different materials

**Fig. 5:**
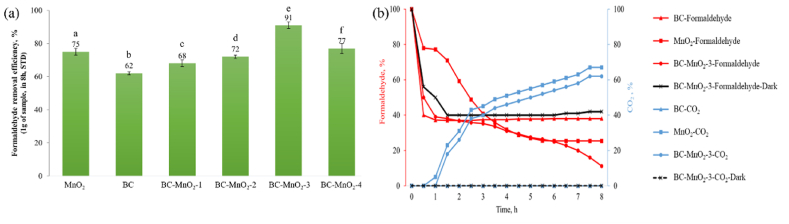
(a) Formaldehyde removal efficiency of the BC, MnO_2_, and BC–MnO_2_ samples and (b) variation of formaldehyde and CO_2_ percentages in the test chamber.

The formaldehyde removal efficiency of BC, MnO_2_, and BC–MnO_2_ samples determined after 8 h is presented in Fig. 5a.

All samples exhibited a specific formaldehyde removal potential. The average values of formaldehyde removal efficiency of the samples were statistically different (P < 0.001, α = 0.05). The BC exhibited a formaldehyde removal efficiency of 62 %. The formaldehyde removal achieved by the BC was attributed to the porous feature of the material, which enabled the physical adsorption of the pollutant assisted by pore-filling mechanisms. Prior research [52] found that the removal of airborne pollutants by BC prepared at 600 °C was controlled by a physical adsorption mechanism where pores in BC served as active sites. The MnO_2_ was capable of removing up to 75 % of the formaldehyde, signifying a promising photocatalytic degradation activity of the synthesized photocatalyst. Doping BC with MnO_2_ further improved its formaldehyde removal efficiency (Fig. 5a). The formaldehyde removal efficiency of BC–MnO_2_ doped particles increased gradually with the increase in MnO_2_ content up to 8 % (in sample BC–MnO_2_-3). However, further loading past 8 % of MnO_2_ (in sample BC–MnO_2_-4) decreased the formaldehyde removal by 15 % compared to BC–MnO_2_-3. At high concentrations, the MnO_2_ impacted the BC's porosity by blocking the pores and cavities, reducing the availability of active adsorption sites. These findings were consistent with the results from physisorption analysis (Table 3), which showed that the porosity of the BC–MnO_2_-4 sample was the lowest amongst all other BC–MnO_2_ samples. Similarly, Dai et al. [23] reported that the formaldehyde conversion capacity of activated carbon fibers- MnO_2_ decreased by 23 % when the MnO_2_ content increased from 16 % to 29 %. They explained that overloading MnO_2_ favored its agglomeration, blocking the pores in the activated carbon fibers' structure and limiting the capture of formaldehyde molecules.

To better understand the formaldehyde removal process, the evolution of formaldehyde and CO_2_ concentrations in the test chamber in the presence of BC, MnO_2_, and BC–MnO_2_-3 samples were plotted as a function of time (Fig. 5b). The formaldehyde concentration dropped sharply and rapidly by up to 60 % for the BC sample in the first 20 min of the test. Then, the adsorption rate slowed down and tended towards stability until the end of the test. At the beginning of the test, the BC pores were free and available, which enabled rapid uptake of the formaldehyde molecules. Subsequently, equilibrium was reached, and BC reached saturation. The saturation phenomenon is common in porous adsorbents. Prior work [16] reported that *Arundo donax*-derived BC reached equilibrium after approximately 20 min of formaldehyde adsorption. It was observed that beyond the equilibrium phase, the formaldehyde removal efficiency was constant and did not further increase. CO_2_ was not detected in the BC sample, indicating that the decrease in formaldehyde concentration was only due to the adsorption mechanism.

For the MnO_2_ sample, the formaldehyde concentration decreased by only 20 % in the first hour of the test. This trend suggested that the MnO_2_ did not adsorb the formaldehyde molecules as fast as the BC sample, which was attributed to the limited porosity of the MnO_2_ (Table 3). As the experimental time progressed, the formaldehyde concentration decreased progressively until the sixth hour of the test. The decrease in formaldehyde concentration was accompanied by a gradual increase in the CO_2_ concentration, signifying that the MnO_2_ was activated and the photodegradation process to convert formaldehyde into CO_2_ started. These results align with prior research [25], which reported that the degradation of formaldehyde by MnO_2_ composite was correlated with CO_2_ generation. The detection of CO_2_ in the test chamber confirmed the successful activation of MnO_2_. During the last 2 h of the experiment, the formaldehyde concentration tended to stabilize, likely because fewer formaldehyde molecules were present in the test chamber, which lowered the chance of being in contact with MnO_2_ particles and continuing the photodegradation process.

For the BC–MnO_2_-3 hybrid sample, the formaldehyde concentration decreased remarkably in the first hour of the test, signifying that the adsorption occurred. However, the adsorption rate was slower than that of the pure BC sample (Fig. 5b), attributed to the differences in porosity between the two samples (Table 3). As the experimental time progressed, the formaldehyde concentration continued to decrease steadily. Simultaneously, CO_2_ production was detected, suggesting that the formaldehyde molecules captured by the BC were being converted by the action of the MnO_2,_ which enabled the adsorption of more formaldehyde. Interestingly, the formaldehyde levels kept decreasing until the end of the test and did not come to stability. These observations suggested that the combined synergetic effect between BC and MnO_2_ improved their formaldehyde removal efficiency. At the beginning of the experiment, adsorption had a dominant role in the formaldehyde removal efficiency. Simultaneously, the decrease in formaldehyde concentration was sharp and rapid. After 1 h, the integrated adsorption-photocatalytic degradation reaction took place. The reduction in formaldehyde concentration was continuous and was accompanied by the generation of CO_2_ as an intermediate of formaldehyde photodegradation. Interestingly, the CO_2_ concentration detected at the end of the test (approximately 60 %) was lower compared to the removed formaldehyde concentration (91 %), which likely informs about the occurrence of other degradation intermediates besides CO_2_. The activity of MnO_2_ was further confirmed by observation of FTIR spectra of the BC–MnO_2_-3 after the formaldehyde removal test (Fig. 4). The spectra exhibited two additional peaks (highlighted with red arrows) compared to the original spectra. The peaks were centered at 2900 cm^−1^ and 2515 cm^−1^ and were attributed to the occurrence of formaldehyde degradation intermediates under the activity of MnO_2_. Similar peaks were observed by Ahn et al. [53] in the spectra of metallic BC after exposure to formaldehyde and were attributed to intermolecular hydrogen bonds of formate and carboxylic acid OH stretch, respectively.

The formaldehyde removal efficiency of the BC–MnO_2_-3 sample was also tested under dark conditions (Fig. 5b). The sample performed almost the same as pure BC. Indeed, the deposited MnO_2_ was not activated without a light source to start the formaldehyde degradation reaction. In this case, the main formaldehyde removal mechanism was adsorption by the BC. Páez et al. [54] investigated the photocatalytic activity of ramsdellite-MnO_2_ via H_2_O_2_ decomposition test in aqueous solution under dark and visible light conditions. They found that the sample's activity under visible light irradiation increased by 120 % compared to activity under dark conditions. They explained that MnO_2_ is a photoactive catalyst and that visible light energy enhanced the electron transfer and photoreduction of Mn^4+^ into Mn^3+,^ resulting in a higher catalytic activity.

#### 3.4.2 Formaldehyde removal mechanism and reusability of BC–MnO_2_

**Fig. 6:**
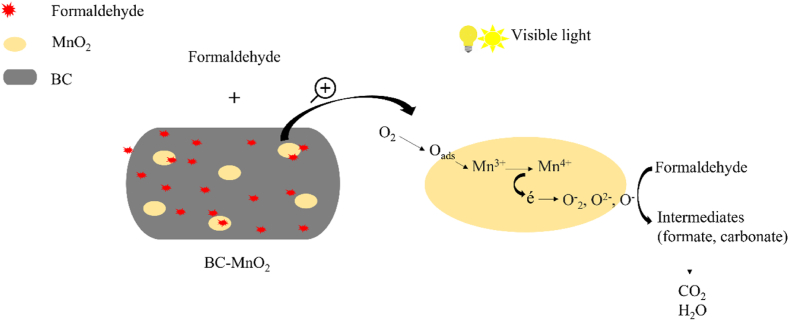
Mechanism of formaldehyde degradation by BC–MnO_2_ particles.

The proposed mechanism of formaldehyde removal by BC–MnO_2_ is represented in Fig. 6.

The mass transfer of formaldehyde molecules from the air phase into the MnO_2_ surface is facilitated by the BC, which exhibited promising adsorption efficiency (Fig. 5). After light absorption and catalyst activation, O_2_ from the surrounding environment was first captured by the oxygen lattices on the MnO_2_ surface to form adsorbed oxygen (O_ads_). The O_ads_ then oxidized the Mn^3+^ into Mn^4+,^ which released electrons to produce oxygen free radicals (O^−^_2_, O^2−^, and O^−^). Subsequently, the formaldehyde molecules were oxidized by oxygen free radicals to create formate and carbonate intermediates [55,56]. These intermediates were further decomposed into CO_2_ and H_2_O, which justifies the generation and increase in CO_2_ concentration as the experiment progressed).

When the formaldehyde removal potential of BC–MnO_2_-3 was investigated for a longer time, the formaldehyde and CO_2_ concentration changes slowed down (Fig. S4a). The formaldehyde removal efficiency of BC–MnO_2_-3 reached 95 % after 24 h. Considering that the experiment was conducted in static conditions, the decrease in removal rate was likely due to the reduction in formaldehyde molecules available in the chamber. The carbon mass balance during the formaldehyde degradation reaction by BC–MnO_2_-3 was determined as a function of time. The percentages of carbon mass balance for formaldehyde and CO_2_ at 0 h, 8 h, and 24 h of reaction are represented in Fig. S4b. The missing part of the carbon balance was labeled as unidentified reaction intermediates. Since the utilized sensors only allowed monitoring of formaldehyde and CO_2_ levels in the test chamber, other intermediates remained unidentified. These reaction intermediates likely consisted of CO and formate [57].

**Fig. 7:**
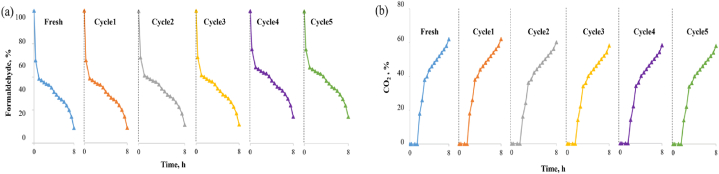
Evolution of the (a) formaldehyde and (b) CO_2_ concentrations during reuse cycles of BC–MnO_2_-3.

The reusability of BC–MnO_2_-3 was assessed by repeating the adsorption-photocatalytic degradation test five times without regeneration, and the results are represented in Fig. 7.

The formaldehyde removal efficiency obtained from fresh, cycle1, cycle2, cycle3, cycle4, and cycle5 was 91 %, 91 %, 89 %, 89 %, 88 %, 87 %, respectively. Hence, the loss of formaldehyde removal efficiency was relatively low. Similarly, the CO_2_ generated during the different cycles remained almost steady. The sample exhibited good stability and formaldehyde removal performance after several uses. Thus, it was utilized to prepare a coating for wooden surfaces.

#### 3.4.3 Depolluting potential of the coatings

**Fig. 8:**
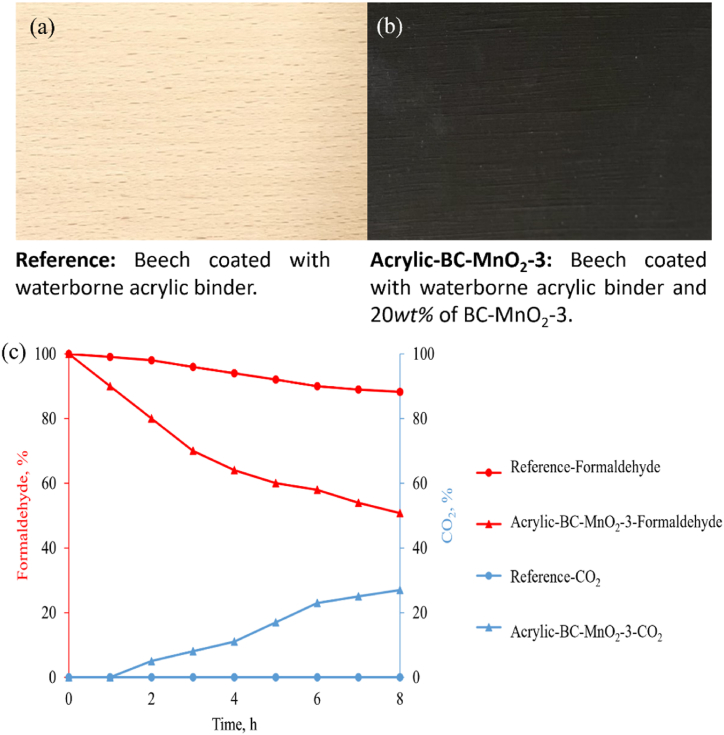
Macroscopic images of the (a) reference and (b) acrylic-BC–MnO_2_-3 coatings on Beech, and (c) variation of formaldehyde and CO_2_ percentages in the presence of the coatings.

The prepared coatings applied on the Beech substrate are represented in Fig. 8a and b. The thickness of the acrylic-BC–MnO_2_-3 coating was 29 ± 7 μm. The variation of formaldehyde and CO_2_ percentages in the presence of the coatings is shown in Fig. 8c.

The total formaldehyd removal efficiency achieved in 8 h was 5 ± 1 % and 43 ± 2 % for the reference and the acrylic-BC–MnO_2_-3 coatings, respectively. For the reference coating, the formaldehyde concentration decreased slightly. This decrease was likely related to the adsorption of some formaldehyde molecules on the surface. The acrylic-BC–MnO_2_-3 coating exhibited higher formaldehyde removal efficiency than the reference due to the integrated adsorption-photocatalytic degradation activity of the BC–MnO_2_-3 particles discussed in section 3.4.1. The decrease of formaldehyde concentration was accompanied by increased CO_2_, indicating formaldehyde conversion (Fig. 8c). The formaldehyde removal capacity per surface area was 1.5 ± 0.2 mg/m^2^ and 6.1 ± 0.4 mg/m^2^ for the reference and the acrylic-BC–MnO_2_-3 coatings, respectively. Findings revealed a promising potential of the developed functional coating as a depolluting composite for indoor environments. However, the coating's formulation needs further optimization and evaluation to determine its efficiency as a coating for wood protection.

## 4 Conclusion

Biocarbon (BC) loaded with MnO_2_ particles was successfully prepared and used to remove formaldehyde indoors via integrated adsorption and photocatalytic degradation technology. Different concentrations of MnO_2_ were investigated, and SEM images revealed that the MnO_2_ tended to settle around and inside the pores of the BC. EDX analysis showed that the manganese contents of the samples ranged between 2 % and 13 %. The extent of disorder in the BC structure increased with the increase in MnO_2_ content, evaluated using the ratio of characteristic bands in the Raman spectra. Peaks corresponding to MnO vibrations were observed in the FTIR spectra of BC–MnO_2_ samples. High loadings of MnO_2_ negatively affected the BC's porosity due to pores blockage, which was reflected by a decrease in the surface area and pores volume. The BC–MnO_2_-3 prepared using a ratio of 1:0.206:0.169 (BC:KMnO_4_:MnSO_4_) resulted in the highest formaldehyde removal efficiency (91 %) and exhibited good stability over reuse cycles. Additionally, the prepared coating showed promising potential for formaldehyde removal in ambient conditions owing to the synergetic effect of BC adsorbent and MnO_2_ photocatalyst. The application of integrated adsorption-photocatalytic degradation technology for designing functional composites with depolluting ability can be promising in providing healthier indoor environments.

## Data availability

Raw data are available in data repository Zenodo: doi.org/10.5281/zenodo.10214401.

## CRediT authorship contribution statement

**Mariem Zouari:** Writing – original draft, Methodology, Investigation, Formal analysis, Conceptualization. **Silvo Hribernik:** Validation, Methodology, Formal analysis. **Laetitia Marrot:** Writing – review & editing, Validation. **Marian Tzolov:** Writing – review & editing, Methodology. **David B. DeVallance:** Writing – review & editing, Supervision.

## Declaration of competing interest

The authors declare that they have no known competing financial interests or personal relationships that could have appeared to influence the work reported in this paper.
