# Supplementary material for: Manganese dioxide-coated biocarbon for integrated adsorption-photocatalytic degradation of formaldehyde in indoor conditions
Source: Heliyon. 2024 Apr 24;10(9):e29993. doi: 10.1016/j.heliyon.2024.e29993 (PMC11061683; doi:10.1016/j.heliyon.2024.e29993)
Supplement: Multimedia component 1 [file mmc1.docx]

**Manganese dioxide-coated biocarbon for integrated adsorption-photocatalytic degradation of formaldehyde in indoor conditions**

Mariem Zouari ^1,2,*^, Silvo Hribernik ^3^ , Laetitia Marrot ^4^, Marian Tzolov ^5^, and David Brian DeVallance ^5^

^1^ InnoRenew CoE, Livade 6a, 6310 Izola, Slovenia

^2^ Faculty of mathematics, natural sciences, and information technologies; University of Primorska, Muzejski trg 2, 6000 Koper, Slovenia

^3^ Faculty of Electrical Engineering and Computer Science, University of Maribor, Koroška cesta 46, SI-2000 Maribor, Slovenia

^4^ FRISSBE, Slovenian National Building and Civil Engineering Institute (ZAG), 1000 Ljubljana, Slovenia

^5^ College of Science and Technology, Commonwealth University, 401 North Fairview Street, Lock Haven, PA 17745, United States

^*^ Corresponding author: mariem.zouari@innorenew.eu

**Table S1.** Preparation of different BC-MnO_2_ samples

| Sample ID | BC, g | KMnO_4_, g | MnSO_4_, g | BC:KMnO_4_:MnSO_4_ ratio |
| --- | --- | --- | --- | --- |
| BC-MnO_2_-1 | 2 | 0.205 | 0.169 | 1:0.103:0.085 |
| BC-MnO_2_-2 | 2 | 0.316 | 0.253 | 1:0.158:0.127 |
| BC-MnO_2_-3 | 2 | 0.411 | 0.338 | 1:0.206:0.169 |
| BC-MnO_2_-4 | 2 | 0.521 | 0.422 | 1:0.261:0.211 |

**Table S2.** Position and ratio of D band and G band in Raman spectra of BC and BC-MnO_2_ samples

| Sample ID | D band position, cm^-1^ | G band position, cm^-1^ | ID/IG |
| --- | --- | --- | --- |
| BC | 1308 | 1573 | 1.22 |
| BC-MnO_2_-1 | 1310 | 1592 | 1.37 |
| BC-MnO_2_-2 | 1313 | 1590 | 1.44 |
| BC-MnO_2_-3 | 1307 | 1588 | 1.47 |
| BC-MnO_2_-4 | 1315 | 1583 | 1.41 |


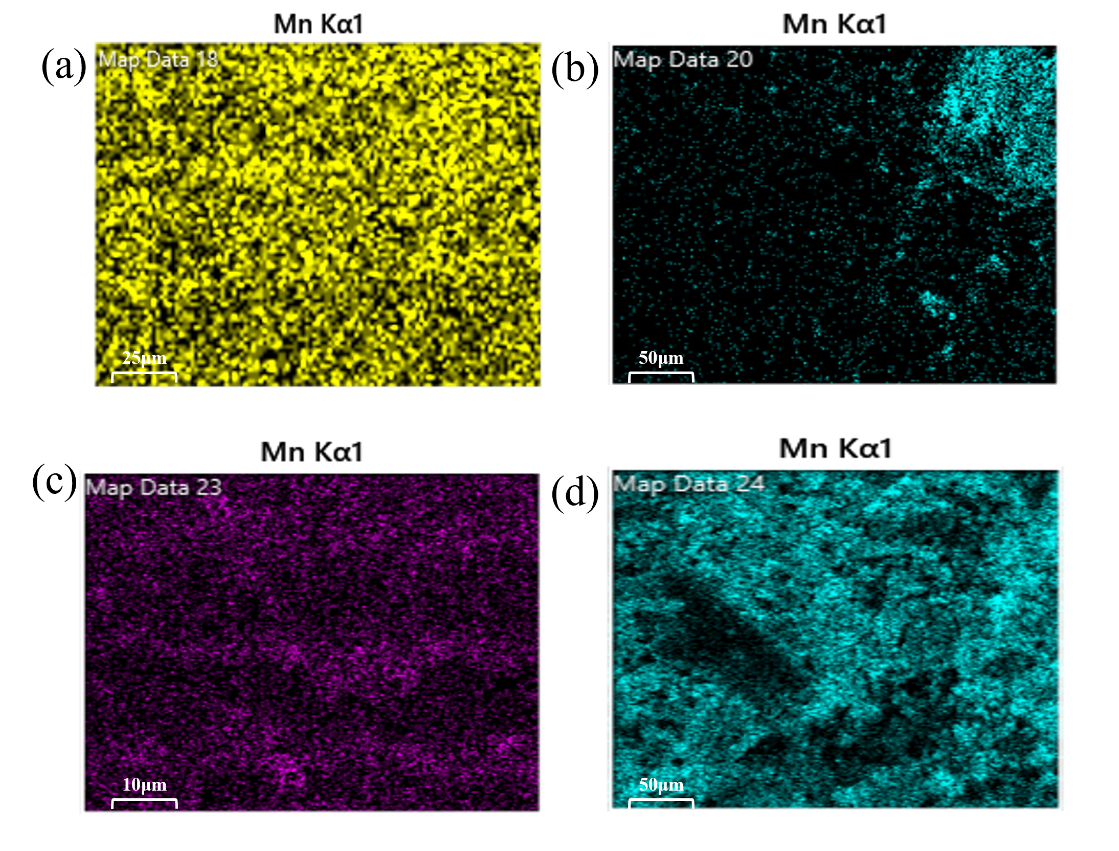


**Fig. S1.** Distribution of the Mn elements in (a) BC-MnO_2_-1, (b) BC-MnO_2_-2, (c) BC-MnO_2_-3, and (d) BC-MnO_2_-4


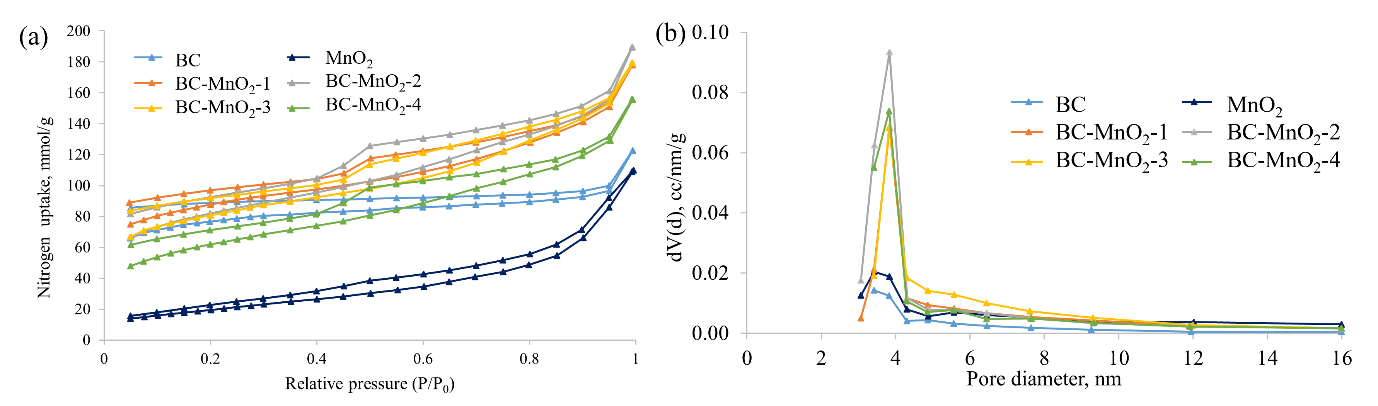


**Fig. S2.** (a) Nitrogen adsorption–desorption isotherms obtained at 77 K and (b) total pores size distributions determined using the BJH model.


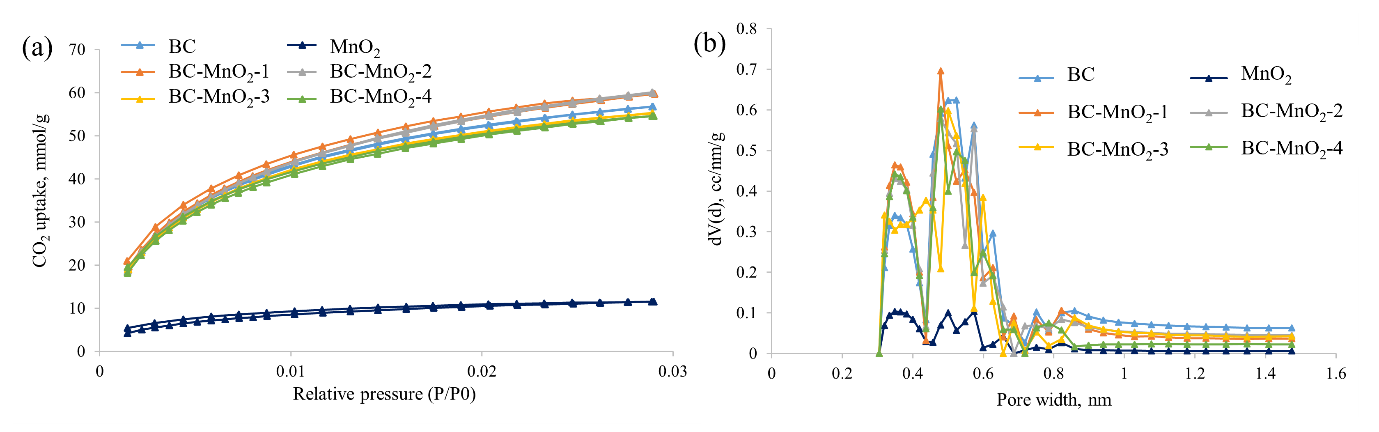


**Fig. S3.** (a) CO_2_ adsorption–desorption isotherms obtained at 273 K and (b) micropores size distributions determined using the DFT model.


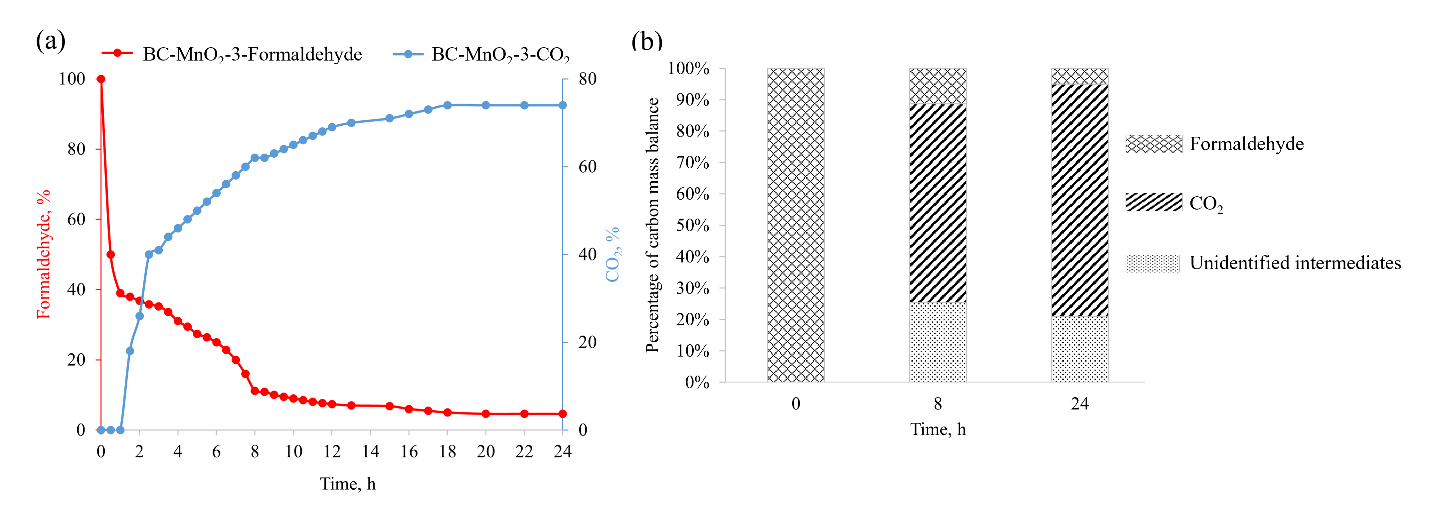


**Fig. S4.** (a) Formaldehyde removal efficiency of BC-MnO_2_-3 in 24h and (b) carbon mass balance.
